# Determination of a Discriminant Dose to Identify Resistance to Amitraz in *Rhipicephalus sanguineus* s.l. (Acari: Ixodidae) from Mexico

**DOI:** 10.3390/insects14070662

**Published:** 2023-07-24

**Authors:** Francisco Martínez-Ibañez, Carlos Cruz-Vázquez, Jorge Osorio-Miranda, Irene Vitela-Mendoza, Leticia Medina-Esparza, Rodolfo Lagunes-Quintanilla, Arturo Chávez-Rodríguez

**Affiliations:** 1 Tecnológico Nacional de México, Instituto Tecnológico El Llano Aguascalientes, Km. 18 Carretera Aguascalientes-San Luis Potosí, El Llano 20330, Aguascalientes, Mexico; pacomtzi@yahoo.com.mx (F.M.-I.); irene.vm@llano.tecnm.mx (I.V.-M.); leticia.me@llano.tecnm.mx (L.M.-E.); 2 Centro Nacional de Servicios en Constatación en Salud Animal, Servicio Nacional de Sanidad, Inocuidad y Calidad Agroalimentaria Carretera Cuernavaca-Cuautla 8534, Col. Progreso, Jiutepec 62550, Morelos, Mexico; joaul_osorio@yahoo.com; 3 Centro Nacional de Investigación Disciplinaria en Salud Animal e Inocuidad, Instituto Nacional de Investigaciones Forestales, Agrícolas y Pecuarias Carretera Cuernavaca-Cuautla 8534, Col. Progreso, Jiutepec 62550, Morelos, Mexico; rodolfo.lagunes@gmail.com; 4 Tecnológico Nacional de México, Instituto Tecnológico de Tlajomulco, Km. 10 Carretera Tlajomulco, Cto. Metropolitano Sur, Tlajomulco de Zuñiga 45640, Jalisco, Mexico; arturo_moisesc@hotmail.com

**Keywords:** brown dog tick, amitraz, discriminant dose, resistance, Mexico

## Abstract

**Simple Summary:**

The brown dog tick is cosmopolitan with medical and veterinary importance. Control with acaricides is the commonly used alternative; however, its indiscriminate use can generate resistance. A discriminant dose (d.d) can be used to rapidly and inexpensively identify resistant populations in samples collected in the field; however, to date, there is limited information on the subject. This study aimed to determine the d.d of amitraz to identify resistance in larvae natives from Mexico and to evaluate its application in field-collected ticks. Because there was no reference strain, the search for samples susceptible to amitraz was carried out in naturally infested rural dogs using a larval immersion test (LIT); the d.d. was determined as a consensus value by multiplying the LC99 × 2, and then, we proceeded to evaluate it in in-field samples by using the LIT technique. The d.d. calculated was 4 ppm. The in-field evaluation found 64% of the samples resistant to amitraz, with mortality percentages between 98.3% and 0.35%; these samples were widely distributed in all the areas of study. With this, bases are established so that scrutiny can be initiated in order to document amitraz resistance in field populations.

**Abstract:**

The brown dog tick, *Rhipicephalus sanguineus* s.l., is considered the most widely distributed three-host tick in the world and has medical and veterinary importance; the control of infestation is carried out with acaricides, towards which it can develop resistance. This study aimed to determine the discriminant dose (d.d) of amitraz to identify resistance in *R. sanguineus* s.l. larvae natives from Mexico and to evaluate its application in field-collected ticks. Engorged ticks were collected from naturally infested dogs residing in rural communities and were incubated for 25 days, and their progeny was used in a larval immersion test (LIT) to be exposed to the d.d. determined in *Rhipicephalus microplus*, and those that were susceptible were analyzed using the LIT in six concentrations. Mortality was analyzed through probit methodology to calculate the lethal concentration (LC) 50 and 99. The d.d. was determined as a consensus value by multiplying the LC99 × 2, and then, we proceeded to evaluate it in in-field samples by using the LIT technique. The d.d. calculated was 4 ppm. The in-field evaluation found 64% of the resistant samples to amitraz with mortality percentages between 98.3% and 0.35%. This dose can be used to rapidly and inexpensively identify resistant populations in samples collected in the field.

## 1. Introduction

The brown tick, *Rhipicephalus sanguineus* s.l. (Latreille) (Acari: Ixodidae), is a cosmopolitan tick with a biological cycle in which three hosts participate, with domestic dogs being the main host; nevertheless, it is capable of parasitizing other domestic and wild animals as well as accidentally parasitizing humans. It is also responsible for the transmission of different pathogens such as *Rickettsia rickettsi*, *R. conorii*, *Babesia canis*, and *Ehrlichia canis*; for this reason, it is considered a tick of medical and veterinary importance. In dogs, it causes different forms of direct damage such as blood loss due to hematophagy, skin injuries due to its bites, and secondary skin infections [1,2]. In Mexico, it is widely distributed, and in the last few years, it has gained importance as a medical and veterinary problem [3,4,5,6].

The acaricides are, up to the present date, the most effective control method against infestation by this tick, while other control measures are of limited application and eventually play a complementary role [7]. Due to the complicated biological cycle of the *R. sanguineus* s.l. [8], in the last few years, the use of long residual effect formulas or the systematic application of acaricides of short persistence has become popular; this can increase the pressure on populations and eventually generate the occurrence of tolerance signs or resistance to molecules, as has been reported in different localities around the world [9,10,11,12,13]. In Mexico, the presence of resistance to different acaricides in the southeast of the country has been reported [14,15]; however, the distribution of this phenomenon in other regions of the country is not yet known.

Amitraz is a contact acaricide derived from formamides with extensive residual action which inhibits the enzymatic system monoamine oxidase, which affects the CNS and kills ticks by inhibiting their nervous system through the blockage of octopamine receptors. The development of resistance is due to mutations in the octopamine/tyramine receptors, and metabolic resistance is caused by the upregulation of genes coding for glutathione-S-transferase and ATP-binding cassette transporters [16]. Amitraz is widely used on the American continent for the control of *R. sanguineus* s.l., and there are several commercial products that contain amitraz alone or combined with other acaricides in different presentations, such as for pour-on application, impregnated in anti-tick collars, and for sprays or immersion baths.

A discriminant dose (d.d) is a single concentration of a pesticide that, applied to a sample in a defined population, will kill a significant proportion of the susceptible genotype, while the resistant genotype will remain alive [17] in such a way that the use of discriminant doses represents a practical alternative, with reduced processing times in the laboratory and low costs to detect the efficacy of acaricides (susceptibility/resistance) in samples collected in the field. With this information, we will be able to establish protocols for their rational use in different regions, as in the case of the cattle tick, *Rhipicephalus microplus* in Mexico [18].

The aim of this study was to determine the discriminant dose (d.d.) to amitraz to identify resistance in *R. sanguineus* s.l. larvae native to Mexico and to evaluate its application in ticks collected from dogs with natural infestation.

## 2. Materials and Methods

### 2.1. Ticks

Engorged ticks were collected from naturally infested dogs, regardless of age, sex, breed/biotype, or size, which were domiciled in different rural communities in the states of Morelos, Tabasco, Tamaulipas, Yucatán, and Veracruz. The dogs had no history of having received any recent treatment with acaricides according to what was stated by their owners, who gave their verbal consent to review and collect ticks from their animals.

A physical inspection of the animals was carried out, which included a detailed review of the head, neck, back, trunk and extremities, interdigital space, and tail, in search of engorged females, which were manually removed and placed in Petri dishes to be transported to the laboratory for taxonomic identification using two different keys [19,20]. The Committee on the Use and Care of Animals of the Instituto Tecnológico El Llano Aguascalientes approved the project (ITEL-CUCA 05/21).

In the laboratory, the engorged females (≥10 mm long) were washed in distilled water and dried by placing them on paper towels. Later, these were placed in individual Petri dishes that were maintained at 28 ± 2 °C and 80–90% relative humidity (RH), in a 12:12 h light/darkness regime until oviposition was completed. After 25 days of incubation, the eggs from each strain were placed in a glass vial and incubated at 28 ± 1 °C, 85–86% RH, and a 12:12 h light/darkness regime to wait for larval hatching.

### 2.2. Bioassays

#### 2.2.1. Diagnosis of Susceptibility

A diagnosis of susceptibility to amitraz was carried out using the d.d. of one Mexican strain of *Rhipicephalus microplus*, toxicologically characterized as a susceptible reference strain [18]. The bioassay used to diagnose susceptibility was the larval immersion test-LIT [21]; the technique uses the commercial presentation of amitraz (Taktic EC 12.5%, MSD Animal Health, Mexico City, Mexico). The test of LIT consists of placing ≈300 larvae in a Petri dish of 15 cm in diameter, between two Whatman No.1 filter papers of 12.5 cm in diameter, adding 10 mL of amitraz d.d. (0.0002%); the control group only received 10 mL of water. The test requires 3 repetitions per group with an immersion time of 10 min. After the immersion time, packages with Whatman filter paper No 1. of 7.5 × 8.5 cm were formed; these were sealed with the help of pressure clips of 55 mm; ≈100 larvae within 15 to 30 days of hatching were previously introduced with a brush. The packages were incubated at 28 ± 2 °C and 80 to 90% relative humidity for 72 h. Subsequently, the packages were reviewed in order to determine the mortality percentages; all larvae that could walk or glide were considered alive, and each treatment was adjusted for non-specific mortality among the untreated controls using Abbott’s formula [22].

#### 2.2.2. Discriminant Dose Determination

The samples that resulted in 100% mortality were considered susceptible to amitraz and were subjected to a second bioassay using LIT methodology as previously described [20] using six concentrations, a control group, and three repetitions in each treatment. The concentrations were as follows: A = 0.0004%, B = 0.0002%, C = 0.0001%, D = 0.00005%, E = 0.000025%, and F = 0.000125%, obtained by 2-fold serial dilutions.

The information generated in the bioassays was subjected to probit analysis using the Polo Plus program (LeOra Software, Petaluma, CA, USA) to calculate the lethal concentrations (LC) 50 and 99, with their respective confidence intervals of 95% and the slope of the regression lines for each population of ticks. Once the LC99 was calculated, the d.d. was determined, which was established as the value of LC99 × 2.

### 2.3. Resistance Evaluation

With the aim of applying the d.d of amitraz in field populations to identify resistance to the molecule, we proceeded to carry out the collection of engorged females of *R. sanguineus* s.l. in naturally infested dogs in urban and rural localities of seven municipalities of the state of Morelos. Using the previously described procedure, 36 samples were obtained and distributed in the following municipalities: Jiutepec (15), Yautepec (10), Cuautla (4), Cuernavaca (2), Temixco (2), Xochitepec (2), and Xoxocotla (1); the management of the samples at the laboratory was the same as the one that was used in the previous stages as well as the conditions of the bioassay using the LIT technique [20], with the peculiarity that the ticks were subjected to the d.d. determined in experiment 2 (Section 2.2.2). We performed three repetitions and one control that was carried out with only distilled water for each sample. The packages were incubated at 28 ± 2 °C and 80 to 90% relative humidity for 72 h. Afterward, the packages were reviewed to determine the mortality percentages. All larvae that could walk or glide were considered alive; a sample was determined as resistant when surviving larvae were found and susceptible when 100% mortality was present in the three repetitions, and each treatment was adjusted for non-specific mortality among the untreated controls using Abbott’s formula [22].

## 3. Results

Identification of the samples with the status of susceptible to amitraz using the d.d determined in *R. microplus* indicated the finding of seven samples with 100% mortality. The state/municipality of origin of the dogs, the sample identification, and the geographical location of the sampling site can be seen in Table 1.

The results of the LIT, focused on determining the d.d. through probit analysis, are shown in Table 2. To calculate the d.d., a total of 14,958 larvae of *R. sanguineus* s.l. were evaluated; the LC50 was 0.0000183 (CI 95% 0.000009–0.0001019), while for LC99, it was 0.000180 (CI 95% 0.000098–0.0004020). The d.d. calculated as the average of all samples evaluated was 4 ppm (0.0004%).

The evaluation results of the resistance in samples taken from naturally infested dogs from the state of Morelos, Mexico, are shown in Table 3. The test allowed us to identify 36% (13/36) of the samples with 100% mortality, which were considered as susceptible to amitraz, while 64% (23/36) were considered resistant; in these samples, mortality was identified in a range between 98.3% and 0.35%. All the municipalities presented resistant samples, and only Cuernavaca did not present susceptible samples. It was not possible to calculate the resistance index of the samples because they did not generate enough larvae for the test.

## 4. Discussion

In recent years, urban societies have increased their affinity with dogs, which provide various benefits to their owners due to the interactions they have with them [23]. The responsible possession of dogs involves various care activities to maintain their health and well-being; an important part of this care has to do with the prevention and control of ectoparasites, mainly fleas and ticks. This activity consists of the application of veterinary formulations with insecticides or ixodicides, whether or not prescribed by the Veterinarian. In many cases, veterinary formulations handled without necessary care in their storage, dosage, application, and frequency of use can be the origin of a poor product response and eventually favor the development of resistance to the compounds [24,25]. The susceptibility or resistance to chemical acaricide compounds in tick populations can be estimated in the laboratory through the use of a d.d. from a sample directly collected from naturally infested dogs, with it being a useful tool that can generate important information in a relatively short period of time and thus facilitate decision-making for the planning, organization, and operation of control programs. In Mexico, the d.d. determined for the routine monitoring of cattle tick resistance is of great practical utility [18].

The brown dog tick, *R. sanguineus* s.l., is a public and veterinary health problem in Mexico, in the tropical and subtropical regions, and is a recurring reason for consultation in veterinary clinics [26]; control is mainly carried out through the application of acaricides in different presentations, but professionally supervised use is not generally present since the sale of these products is not regulated and any person can have access to them, increasing the probability of wrong use, underdosing, and/or indiscriminate use, which can lead to resistance development or control infestation failure.

Under ideal conditions, for d.d. determination, it is recommended to have a susceptible strain as a reference, preferably native to the region; unfortunately, in Mexico, there is not an *R. sanguineus* s.l. strain with such characteristics. For this reason, a search for susceptible ticks was carried out in rural communities, where access to and the use of acaricides is limited; hence, lower selection pressure was expected, and therefore, the success possibilities were higher and the results were more reliable. For this purpose, numerous collections and tests were performed using the d.d. recommended for *R. microplus*, achieving the identification of seven susceptible samples in which a d.d. of 4 ppm was determined (Table 2); to the best of our knowledge, this is the first amitraz d.d determined in *R. sanguineus* s.l. populations in Mexico.

Amitraz began to be used in Mexico in 1984 to control cattle ticks, with its use increasing from the 1990s as an alternative to treat populations resistant to organochlorines, organophosphates, and pyrethroids; however, in the year of 2002, the first case of resistance in *R. microplus* was reported [21]. In the case of *R. sanguineus* s.l., the widespread presence of amitraz resistance in samples collected from dogs of different communities in the state of Yucatán was reported; 85.7% of the populations were classified as resistant, and low inter-population variation in the phenotypic level of resistance was observed [14]; in another study developed in the municipality of Irapuato, Guanajuato, no resistant cases were identified upon exposing the larvae of *R. sanguineus* to different concentrations of the commercial product [27].

In the present study, the application of the d.d. (4 ppm) allowed for the identification of 64% of the samples analyzed as amitraz resistant, showing that for this characteristic in ticks collected in all the municipalities included in the study, this value is lower than the reported in the state of Yucatán, but the high percentage of resistant samples is still alarming. The mortality percentages registered under 100% had wide variation, but it can be estimated that the detected resistance is more important in two populations: Yautepec 3 (0.35% mortality) and Jiutepec 3 (56.5% mortality), which represents 9% of the samples considered as resistant; the rest of the samples had mortality rates between 98.3 and 75.2% (Table 3), which, from our point of view, indicates low susceptibility to amitraz, which may be generating failures in the control of infestations. However, according to Suraj et al. [28], it can be considered that mortality < 51% indicates high resistance. Unfortunately, it was not possible to calculate the resistance index; however, the evidence generated with the challenge to the d.d. (4 ppm) is enough to consider that the phenomenon of resistance is present in a high proportion of the samples analyzed, which also suggests that these populations have been exposed to the acaricide on a recurrent basis.

The presence of amitraz resistance in other parts of the world is poorly documented. The literature refers to the fact that in different Spanish populations, resistance to this acaricide has not been detected [10], while in Cuba, resistance to the strain “Bejucal 2010” was identified [13]. In another study, resistance to a strain collected from the Corozoal Army Veterinary Quarantine Center in Panama was detected, as well as the strain “Center Point” collected in Kerrville, Texas [9].

The use of the d.d. of different acaricides in the monitoring of susceptibility to molecules is a tool of high technical–scientific value, which can assist in the fast diagnosis of resistance and help in decision-making in reference to infestation control; plus, it requires a few full ticks to develop the test. The resistance index calculation can provide more specific data about the phenomenon magnitude, but it is a process that requires a larger full tick number and more time and is more expensive.

Preventing the development of tolerance/resistance is a complex task in which several actors must intervene, including veterinarians, dog owners, pest controllers, the veterinary pharmaceutical industry, and health authorities, so that each one collaborates harmoniously to carry out correct handling of the chemical control of *R. sanguineus* s.l with the aim of giving better use to this alternative, which should include the rotation of acaricides and environmental control under adequate knowledge of the biological cycle and the seasonality of the infestation in the different geographical areas.

## 5. Conclusions

The present study has allowed for the determination of an amitraz d.d in samples of *R. sanguineus* s.l. ticks collected in rural areas of Mexico, which was used in the study to detect resistance in tick populations that naturally infest dogs in the state of Morelos, Mexico, and which has allowed us to document the high frequency of samples resistant to the acaricide. With this, bases are established so that scrutiny can be initiated in order to document amitraz resistance in populations of *R. sanguineus* s.l. in the country, in addition to providing a reference for the international scientific community. The rational control of *R. sanguineus* s.l. infestations using acaricides will allow us to limit resistance development, as well as the negative effects on dogs’ health, their owners health, and public health.

## Figures and Tables

**Table 1:** The geographical location of the naturally infested dogs from which the *R. sanguineus* s.l. larvae susceptible to amitraz were collected.

State/County	Sample	Longitude *	Latitude *
Morelos/Jiutepec	Mor 3	−99.1552788	18.895000
Morelos/Jiutepec	Mor 5	−99.17134000	18.89251100
Morelos/Jiutepec	Mor 6	−99.153333	18.880000
Morelos/Jiutepec	Mor 9	−99.1833	18.1833
Morelos/Jiutepec	Mor 12	−99.153333	18.880000
Morelos/Jiutepec	Mor 13	−99.067	18.883
Veracruz/SJ. Evangelista	Ver 19	−95.1293	17.8817

* Geographical coordinates in standard decimal degrees.

**Table 2:** Lethal concentration (LC) 50 and 99 of amitraz and d.d. of total samples in *R. sanguineus* s.l. from rural areas using probit analysis.

Sample	*n*	Slope	Chi-Square	LC50 (95% CI) ^a^	LC99 (95% CI) ^a^
Mor 3	2153	2.250	106.93	0.0000337 (0.0000082–0.0000185)	0.0001149 (0.0000723–0.0002948)
Mor 5	2292	1.426	333.81	0.000009 (0.0000001–0.0000173)	0.000385 (0.00013–0.0003488)
Mor 6	2313	3.049	89.24	0.0000322 (0.0000276–0.000372)	0.0001864 (0.0001364–0.0002954)
Mor 9	2216	2.464	114.17	0.0000226 (0.0000172–0.0000283)	0.0001985 (0.0001249–0.0004388)
Mor 12	1967	3.009	54.01	0.00001456 (0.00001135–0.0000175)	0.000086 (0.0000607–0.0001548)
Mor 13	2067	1.829	95.65	0.0000091 (0.0000034–0.000143)	0.0001706 (0.0000971–0.0006221)
Ver 19	1950	1.895	136.75	0.000007 (0.0000017–0.000117)	0.0001187 (0.0000649–0.0006593)
Ʃ	14,958	15.92	930.56	0.00012816 (0.00006955–0.0007136)	0.0012601 (0.0006863–0.002814)
mean	2136.86	2.27	132.94	0.0000183 (0.0000099–0.0001019)	0.000180 (0.0000980–0.0004020)
					d.d. 0.0004 (4 ppm)

n = Total number of tick larvae used for the test. ^a^ = The values represent the percentage of active ingredient (*w*/*v*) applied.

**Table 3:** Mortality in *R. sanguineus* larvae in 36 samples from naturally infested dogs in the state of Morelos, Mexico, challenged with an amitraz d.d. (4 ppm).

Municipality/	Mortality	Municipality/	Mortality
Sample	(%)	Sample	(%)
Temixco 1	98.0	Jiutepec 1	100
Temixco 2	100	Jiutepec 2	82.1
Cuernavaca 1	75.2	Jiutepec 3	56.5
Cuernavaca 2	81.1	Jiutepec 4	81.9
Yautepec 1	75.5	Jiutepec 5	98.3
Yautepec 2	85.1	Jiutepec 6	93.7
Yautepec 3	0.35	Jiutepec 7	84.8
Yautepec 4	100	Jiutepec 8	91.9
Yautepec 5	100	Jiutepec 9	100
Yautepec 6	100	Jiutepec 10	100
Yautepec 7	92.4	Jiutepec 11	92.5
Yautepec 8	79.1	Jiutepec 12	100
Yautepec 9	92.5	Jiutepec 13	100
Yautepec 10	100	Jiutepec 14	87.5
Cuautla 1	94.7	Jiutepec 15	75.2
Cuautla 2	94.9	Xochitepec 1	100
Cuautla 3	100	Xochitepec 2	97.1
Cuautla 4	95.7	Xoxocotla 1	100

## Data Availability

Data are available from the authors upon request.

## References

[1] Dantas-Torres F. (2008). The brown dog tick, *Rhipicephalus sanguineus* (Latreille, 1806) (Acari: Ixodidae): From taxonomy to control. Vet. Parasitol..

[2] Dantas-Torres F., Otranto D. (2015). Further thoughts on the taxonomy and vector role of *Rhipicephalus sanguineus* group ticks. Vet. Parasitol..

[3] Cruz-Vázquez C., García-Vázquez Z. (1999). Seasonal distribution of *Rhipicephalus sanguineus* ticks (Acari: Ixodidae) on dogs in an urban area of Morelos, Mexico. Exp. Appl. Acarol..

[4] Álvarez-Hernández G., González-Roldán J.F., Hernández-Milan N.S., Ryan L.R., Behravesh C.B., Paddock C.D. (2017). Rocky Mountain spotted fever in Mexico: Past, present, and future. Lancet Infect. Dis..

[5] Galindo-Velasco E., Cruz-Vázquez C., Díaz-Chapula H., Lezama-Gutiérrez R., Chan-Copul W. (2020). Annual Infestation Pattern of *Rhipicephalus sanguineus sensu lato* on naturally infested dogs in a tropical sub-humid region of Mexico. Southwest. Entomol..

[6] Sánchez-Montes S., Salceda-Sánchez B., Bermúdez S.E., Aguilar-Tipacamú G., Ballados-González G.G., Huerta H., Aguilar-Domínguez M., Mora J.D., Licona-Enríquez J.D., Mora D.D. (2021). *Rhipicephalus sanguineus* complex in the Americas: Systematic, genetic diversity, and geographic insights. Pathogens.

[7] Pereira C.P.M., Oliveira P.R., Furquim K.C.S., Bechara G.H., Camargo-Mathias M.I. (2009). Effects of fipronil (active ingredient of Frontline 1) on salivary gland cell of *Rhipichepalus sanguineus* females (Latreille 1806) (Acari: Ixodidae). Vet. Parasitol..

[8] Dantas-Torres F. (2010). Biology and ecology of the brown dog tick, *Rhipicephalus sanguineus*. Parasites Vectors.

[9] Miller R.J., George J.E., Guerrero F., Carpenter L., Welch J.B. (2001). Characterization of acaricide resistance in *Rhipicephalus sanguineus* (Latreille) (Acari: Ixodidae) collected from the Corazal army veterinary quarantine centre, Panama. J. Med. Entomol..

[10] Estrada-Peña A. (2005). Etude de la résistance de la tique brune du chien, *Rhipicephalus sanguineus* aux acaricides. Rev. Vet. Med..

[11] Borges L.M.F., Soares S.F., Fonseca I.N., Chaves V.V., Louly C.C.B. (2007). Resistencia acaricida em larvas de *Rhipicephalus sanguineus* (Acari: Ixodidae) de Goiânia-GO. Rev. Patol. Trop..

[12] Eiden A.L., Kaufman P.E., Oi F.M., Allan S.A., Miller R.J. (2015). Detection of permethrin resistance and fipronil tolerance in *Rhipicephalus sanguineus* (Acari: Ixodidae) in the United States. J. Med. Entomol..

[13] Encinosa-Guzmán P.E., Bello-Soto Y., Rodríguez-Mallon A. (2016). Genetic and biological characterization of a Cuban tick strain from *Rhipicephalus sanguineus* complex and its sensitivity to different chemical acaricides. Int. J. Acarol..

[14] Rodríguez-Vivas R.I., Ojeda-Chi M.M., Trinidad-Martínez I., Bolio-González M.E. (2016). First report of amitraz and cypermethrin resistance in *Rhipicephalus sanguineus* sensu lato infesting dogs in Mexico. Med. Vet. Entomol..

[15] Rodríguez-Vivas R.I., Ojeda-Chi M.M., Trinidad-Martínez I., Pérez de León A.A. (2017). First documentation of ivermectin resistance in *Rhipicephalus sanguineus* sensu lato (Acari: Ixodidae). Vet. Parasitol..

[16] Jonsson N.N., Klafke G., Corley S.W., Tidwell J., Berry C.M., Koh-Tan H.C. (2018). Molecular biology of amitraz resistance in cattle ticks of the genus *Rhipicephalus*. Front. Biosci..

[17] Ffrench-Constant R.H., Roush R.T., Roush R.T., Tabashnik B.E. (1990). Resistance detection and documentation: The relative roles of pesticidal and biochemical assays. Pesticide Resistance in Arthropods.

[18] Martínez I.F., Miranda M.E., Jasso V.C.E., Cossio B.R., Kumar S., Cossio-Bayugar R., Kumar S.A., Miranda-Miranda E., Kumar C.A. (2021). Reference tick strains as an important biological material for acaricide resistance characterization. The Entomological Guide to Rhipicephalus.

[19] Barros-Battesti D.M., Arzua M., Bechara G.H. (2006). Carrapatos de Importância Médico-Veterinária da Região Neotropical: Um Guia Ilustrado para Identificação de Espécies.

[20] Martínez I.F., Rodríguez-Vivas R.I. (2015). Garrapatas de importancia veterinaria. Técnicas para el Diagnóstico de Parásitos con Importancia en Salud Pública y Veterinaria.

[21] Soberanes C.N., Santamaria V.M., Fragoso S.H., García V.Z. (2002). Primer caso de resistencia al amitraz en la garrapata del ganado *Boophilus microplus* en México. Tec. Pecu. Mex..

[22] Abbott W.S. (1925). A method for computing the effectiveness of an insecticide. J. Econ. Entomol..

[23] Wood L., Giles-Corti B., Bulsara M. (2005). The pet connection: Pets as a conduit for social capital?. Soc. Sci. Med..

[24] Blagburn B.L., Dryden M.W. (2009). Biology, treatment and control of flea and tick infestations. Vet. Clin. N. Am. Small Anim. Pract..

[25] Coles T.B., Dryden M.W. (2014). Insecticide/acaricide resistance in fleas and ticks infesting dogs and cats. Parasites Vectors.

[26] Cruz-Vázquez C., Quiroz-Romero H., Ibarra-Velarde I. (2006). Ixodidosis. Enfermedades Parasitarias en Perros.

[27] Hernández-Herrera O.G., Arriola-Mosqueda L.A., Prieto-Avella E.C., Jiménez-Lara Y., Lazcano-Ortíz L., Angel-Sahagún C.A., Valencia-Posadas M., Gutiérrez-Chávez A.J., Cruz-Vázquez C. (2016). Evaluación de acaricidas sobre *Rhipicephalus sanguineus* (Latreille) (Acari: Ixodidae). Entomol. Mex..

[28] Suraj R.-A., Rambarran R., Ali K., Harbajan D., Charles R., Sant C., Georges K., Suepaul S. (2019). A comparison of the efficacy of two commercial acaricides (fipronil and amitraz) with *Azadirachta indica* (neem) on the brown dog tick (*Rhipicephalus sanguineus*) from canines in Trinidad. Transbound. Emerg. Dis..

